# The Diagnosis of Chancre—Prognosis of Treatment

**Published:** 1892-07

**Authors:** C. G. Darling


### COMMUNICATIONS.

### The Diagnosis of Chancre.—Prognosis of Treatment.

BY C. G. DARLING, M. D.
Clinical Lecturer on Oral Pathology and Surgery in the College of Dental Surgery.

It is a matter of great importance to your patient that no mistake is made in the diagnosis of the initial lesion; this while commonly easy is sometimes difficult, you may see the chancre in an early stage of development, when the corresponding lymphatics are not enlarged to any appreciable degree; an innocent looking chancre may be the forerunner of a most malignant form of syphilis, a pin scratch may look far more dangerous than the lesion present, but the mistake usually made is, that the abrasion is an innocent sore, and will soon pass away. The lesion rapidly assumes its characteristic form, the friends which accompany it on its course make their appearance. The base of the primary sore begins to harden and the neighboring lymphatics become enlarged. All other signs may be delusive, but the peculiar hardness of the base is a certain sign to one familiar with the disease, and he rarely makes a mistake in diagnosis. You will carefully watch the course of the suspected lesion for about two weeks when you will find the glands associated with it are enlarged.

You must distinguish chancre from ordinary fissure of the lip, from herpes and epithelioma, the time and course of formation will be a great aid in diagnosis, with fissure there is no induration or enlargement of glands, while herpes rarely comes as a single sore, epithelioma is far more painful and the induration of neighboring glands does not begin until the disease is far advanced. Epithelioma is a disease of advanced age, syphilis appears in early life.

### PROGNOSIS.

Chancre is not a serious lesion, unless complicated. When situated on the tongue there may be pain and difficulty in mastication. Ory quotes the case of a young girl with chancre on the upper lip, which became phagedenic, destroying a large portion of the lip; she had repeated hemorrhages, and it was only by the greatest care that her life was saved. Young persons who are well nourished and free from hereditary taint have little to fear if the lesion can be kept aseptic. The early excision of chancre has of late been recommended as good treatment when the lesion is so located that the expedient can be adopted. I can conceive of no more favorable location for carrying out this treatment, than in chancre of the lip; not only may the danger of general infection be lessened but the danger of transmitting the disease to others is for a time removed. Peroni claims to have destroyed the initial lesion by electrolysis, successfully aborting twenty-one out of twenty-nine cases. The treatment was applied before the glands became enlarged, or within seven days after the appearance of the sore. De Ehlers found while employing this treatment that the course while not always aborted was far less malignant. He strongly favors early excision. If there is a bacillus of syphilis I see no reason why we might not, by excision, expect to limit the dosage of the virus if not entirely destroy it; the wound usually tends to heal, leaving but a small cicatrix behind. When the lesion is in a locality that will not admit of excision, as within the mouth or on the tongue, it may be treated by using non-irritating antiseptic washes.

The enlarged lymphatics which accompany chancre need no special treatment, they are painless, not being noticed by the patient until his attention is called to them. This condition disappears after three or four weeks by resolutions, though like the induration of chancre, they may remain enlarged for six or eight months, rarely do the glands tend to suppurate unless they have been injured.

### THE SECONDARY STAGE OF SYPHILIS.

This stage is marked by constitutional changes and cutaneous lesions, there is usually fever, headache, pains in the muscles and joints, neuralgia, insomnia, etc., in the begining of the secondary stage. Syphilitic fever, however slight, plays an important part in the history of the case; it begins about fifty to seventy days after infection, or three to five weeks after the appearance of the chancre, it is usually preceded by headache and chill, it may so closely resemble intermittent fever as to be mistaken for it, and, while the course of the fever may be quite severe, the functions of the body are but little disturbed; it pursues no definite course, but the prolonged types are frequently followed by severe general lesions. Quinine has no influence on its course, but it yields readily to the proper use of mercury. Neuralgic pains may be felt in various parts of the body, but frequently come in the frontal and supraorbital branches of the facial nerve, it is usually worse at night. The lingual, auricular, mastoid and great occipital, are the nerves of the head most frequently involved. Neuralgia may for a long time be the only recognized lesion of syphilis, hence it is always well to employ mercury in the treatment of stubborn and mysterious forms of neuralgia, the headache of early syphilis is deepseated; it may be in the frontal, temporal, or occipital portion, and may be severe enough to cause loss of sleep or prevent mental occupation. The skin and mucous membrane are easily irritated, wounds and scratches do not heal readily, the sense of touch of heat and cold may be impaired or entirely lost, this condition may be restricted to certain portions of the body, as a portion where the sensation depends on the activity of a certain nerve, or it may extend over the whole body. These conditions are rare, but may last several months, and are most common to the cheeks and mammary region.

### SKIN LESIONS.

The various eruptions which appear on the skin are called syphilides or syphilodermata, and are caused by cell infiltration, and localized hyperæmia, they possess certain characteristics which serve to distinguish them from other affections of the skin. It is a common condition to find several varities of skin lesions present at the same time, some will be erythematous, simple, red, non-elevated patches of various size and shape, others will be vesicular, some will be moist, while others are dry and scaly, at other times they may be quite uniform in character. Their very peculiar color attracts the attention of the observer, this assumes a particular shade according to the age and stage of their existence, at first they are pinkish red, but gradually fade to brownish red, precisely the shade of lean ham. The copper color is peculiar to those lesions which appear on the face, while the pink shades appear where the body is covered, particularly on the extremities, these changes in color are probably due to deposit of the coloring of the blood in the spots affected. There may be relapses after the spots have wholly or in part disappeared. The peculiar rounded form or arrangement of the eruption is of great importance in diagnosis, especially when it is quite persistent. Skin and mucous lesions are generally round when found alone, and when many are present at the same time, they may assume a crescent form or a part of a circle; this circumstance alone does not determine the nature of the disease, but is a valuable aid to diagnosis when the case is obscure. It is characteristic of these eruptions to be indolent, and they may remain weeks or months without any change. They may be without sensation, no itching except at that time when the eruption makes its first appearance.

### SYPHILITIC ERYTHEMA.

This is the earliest and most common of all the syphilitic skin lesions, it is said to exist in all cases, though in some instances so mild as to escape observation, because it is confined to the parts of the body which are covered by the clothing, it makes its appearance about the sixth or eighth week after the development of the chancre, but may come much later if its appearance has been retarded by the administration of mercury.

Syphilitic Erythema, appears in small oval spots, they may also be irregular, from a line to half an inch in diameter, sometimes a number of these will run together and form a patch. When the lesions first make their appearance, they are of a pale rose color, but may change to red or purple, the cause of this change is due to hyperæmia, the skin capillaries being engorged with blood. In some cases there is only slight mottling of the skin which rapidly disappears; exposure to cold or undressing in a cool room will bring the spots out distinctly.

The eruption usually appears first on the abdomen, chest and back, it is rarely observed on the face, but may persist for a long time on the hands and the soles of the feet, when they are invaded. No other skin lesion is of such diagnostic value as this, when it appears in the palms of the hands; here the lesions soon begin to form scales, which are continuously thrown off for some time. The same is true of the lesions appearing on the face, because these parts of the body are exposed. Other lesions may come with the eruption, the hair may fall out, the nails become dry and scaly, and the eye may be subject to inflammatory conditions. Superficial ulceration may take place, where the surfaces of the skin rub together, and from it may be found a foul secretion.

The course of erythematous lesions is slow; when once fully developed it may remain unchanged for a long time, its persistence depends upon the degree of hyperæmia present and the treatment employed. Relapses are common and it may become a mixed eruption, however, it gradually disappears, pigmentation left by hyperæmia is gradually absorbed, and the time comes when the skin shows no trace of the former lesions. This eruption may be mistaken for measles, scarlatina, or that produced by the admininstration of certain drugs, as cubebs, sandalwood, and mercury. The history will generally make this clear, the eruptions of measles and scarlet fever are fleeting, and accompanied by more fever than usually attends syphilitic eruptions.

### PAPULAR SYPHILIDES.

Usually small and conical; they may appear in groups or singly. When the hand is passed over them, slight elevations are felt, the summits of which, become covered with minute scales. When they first appear they are bright red, then change to a dusky color, this change being characteristic of nearly all syphilitic eruptions. This form of eruption makes its appearance about the third or fourth month of infection, is chronic, obstinate in its course, and subject to relapses. There are large papular syphilides, which begin as small spots and rapidly increase in size until they may be an inch in diameter, they are scaly, elevated, and well defined, changing in time from a red to a coppery hue, they are chronic in their course and do not readily disappear, their favorite location is about the mouth or nape of the neck, this is a common syphilitic skin lesion, occuring at any stage of the disease, but usually seen just before or just after the general eruption. Once formed they may remain for weeks, but yield readily to treatment. In their course they may become moist papules, either upon the general surface of the skin or about the mouth ; a thin crust is formed by the accumulation and drying of the moisture, which is thrown off as scales or remains as a scab, under which healing takes place, the scab drops off leaving a large coppery-colored scar. When situated in a locality where there is much irritation by surfaces rubbing together the discharge is muco-pus, and of a disgusting odor.

Another variety of the moist papular syphilide occurs frequently just within the mouth, they may also be seen between the folds of the fingers, where they take the form of fissures. There is a squamous or scaly form appearing on the hands and feet, occasionally on the face, which is of great value in diagnosis, and is very peristent, it may appear at any stage of the disease after the general eruption. There is no itching or pain and the proper treatment rapidly limits its course. When first seen the papules are elevated, sharply defined, deep red in color; after a time these well defined elevations tend to run together forming a general scaly surface, which may thicken, crack, and persist if no treatment is used; the disease may extend even to the nails making them brittle and thickened.

Essence of Cassia as an Antiseptic.—Dr. Black, *La Semaine Medicale*, No. 59, 1891, has found the essence of Chinese cassia a powerful antiseptic, even in a 1 : 4,000 solution.

It is superior to boric and carbolic acid ; it is not irritating, and has an agreeable odor. It may be used with advantage as an antiseptic in surgical and gynecological practice, and as an emulsion, dissolved in distilled water, or mixed with boric acid. For concentrated solutions use a concentrated solution in cinnamon water.

